# A Recurrent and Atypical Form of Guillain-Barré Syndrome: A Case Report

**DOI:** 10.7759/cureus.113362

**Published:** 2026-07-25

**Authors:** Larbi Ed Dafali, Shaimae Semlali, Hind Lachraf, Saad Elharrak, Salma Ech Cherif El Kettani

**Affiliations:** 1 Anesthesia and Intensive Care, Children's Hospital of Rabat, Rabat, MAR; 2 Anesthesia and Critical Care, Children's Hospital of Rabat, Rabat, MAR; 3 Neurology, Children's Hospital of Rabat, Rabat, MAR

**Keywords:** child, descending paralysis, diagnosis, guillain-barré syndrome, recurrence

## Abstract

Guillain-Barré syndrome (GBS) is an acute inflammatory polyradiculoneuropathy characterized by rapidly progressive sensorimotor impairment that is classically ascending and monophasic. Recurrent and descending forms are rare and may complicate diagnosis. We report an atypical recurrent case of GBS in a child presenting with descending paralysis. We describe a nine-year-old girl with a history of typical GBS who was admitted to the pediatric ICU for acute descending flaccid paralysis. The clinical course rapidly progressed to tetraplegia with bulbar and respiratory involvement. Electroneuromyography showed acute demyelinating motor polyneuropathy, CSF analysis revealed albuminocytologic dissociation, spinal MRI demonstrated cauda equina nerve root enhancement, and anti-ganglioside antibodies were positive. The patient required mechanical ventilation and multiple courses of IVIG, with subsequent significant neurological recovery and complete remission at three months. Recurrent GBS is rare. Descending paralysis is atypical in this disease and may delay diagnosis. In such cases, repeated clinical evaluation and supportive paraclinical investigations are essential. The benefit of repeated courses of IVIG in poorly responsive cases remains uncertain and is supported by limited evidence. This case highlights the importance of considering GBS in acute flaccid paralysis, even in atypical and recurrent presentations, and underscores the diagnostic value of paraclinical investigations in such contexts.

## Introduction

Guillain-Barré syndrome (GBS) is the most frequent cause of acute flaccid paralysis, affecting approximately 0.4-2.4 per 100,000 persons per year [1]. It is an acute polyradiculoneuritis characterized by rapidly progressive sensorimotor involvement, generally ascending and monophasic [2,3]. We present the case of a nine-year-old child admitted to the ICU of the Children’s Hospital of Rabat for GBS with two rare features: recurrence and the descending nature of the paralysis, which made the diagnosis particularly challenging. In this context, the Brighton criteria, a standardized diagnostic certainty classification system for GBS [4], may represent a valuable tool for supporting diagnosis in atypical presentations and reducing diagnostic uncertainty.

## Case presentation

A nine-year-old girl with normal psychomotor development and up-to-date vaccinations was admitted to the pediatric ICU. Her medical history was notable for a previous episode of typical GBS (ascending paralysis) at the age of two years, which required treatment with IVIG and rehabilitation, leading to complete recovery after four months.

In July 2025, she presented with an influenza-like illness associated with transient diarrhea. Fifteen days later, she developed a symmetrical motor deficit affecting both upper limbs that progressed over four days. Upon admission to the Children’s Hospital of Rabat, the patient was conscious and afebrile, with symmetrical weakness of both upper limbs graded at 3/5. There were no sensory disturbances, cranial nerve involvement, or sphincter dysfunction.

**Figure 1:**
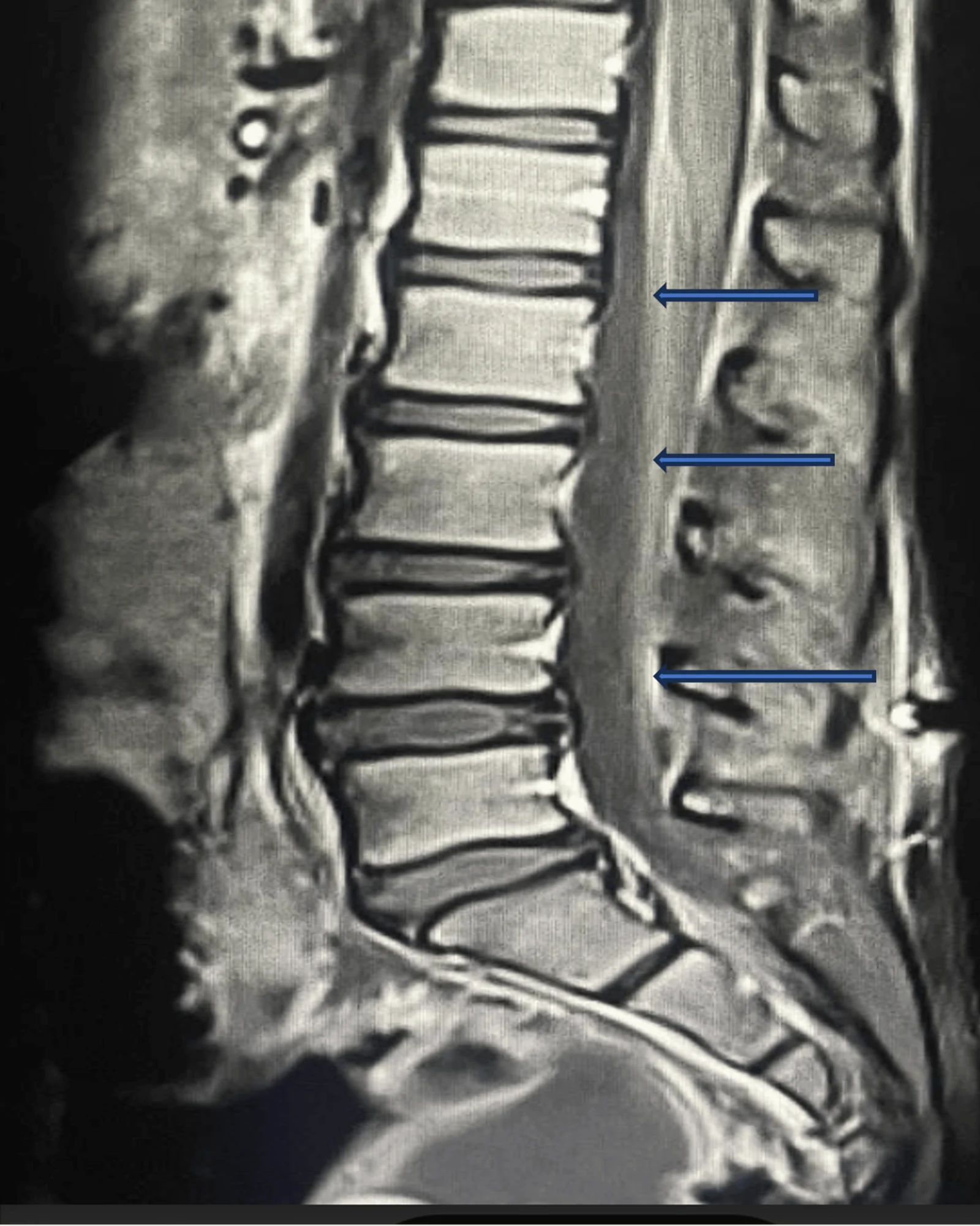
Spinal MRI showing enhancement of the cauda equina nerve roots (blue arrows), a sign of radiculoneuritis

Within 24 hours, her condition worsened, with the development of flaccid tetraplegia, dysphagia, loss of the cough reflex, hypoventilation, and generalized areflexia. Electroneuromyography revealed findings consistent with acute demyelinating motor polyneuropathy. CSF analysis performed at admission was normal; however, a second lumbar puncture on Day 5 demonstrated albuminocytologic dissociation (Table 1). Spinal MRI showed enhancement of the cauda equina nerve roots (Figure 1).

**Table 1:** Results of laboratory investigations

Parameter	Result	Units	Reference values
CSF (initial lumbar puncture)			
Protein level	0.20	g/L	0.15-0.45 g/L
Glucose level	0.80	g/L	≈60% of blood glucose
Cell count	0	cells/mm³	<5
CSF (Day 5)			
Glucose level	0.70	g/L	≈65% of blood glucose
Cell count	0	cells/mm³	<5
Infectious serologies			
HIV	Negative	-	Negative
Hepatitis B	Negative	-	Negative
Hepatitis C	Negative	-	Negative
Epstein-Barr virus	Negative	-	Negative
Cytomegalovirus	Negative	-	Negative
Syphilis	Negative	-	Negative
*Borrelia burgdorferi*	Negative	-	Negative
Stool culture for poliovirus	Negative	-	Negative
Botulism investigations			
Botulinum toxin assay (serum)	Negative	-	Negative
Botulinum toxin assay (stool)	Negative	-	Negative
Stool culture for* Clostridium botulinum*	Negative	-	Negative
Anti-ganglioside antibodies			
Anti-GT1a IgG	Strongly positive	-	Negative
Anti-GQ1b IgG	Strongly positive	-	Negative

Anti-ganglioside antibody testing, performed on the second day of admission to the ICU, revealed strongly positive IgG anti-GT1a and anti-GQ1b antibodies. An extensive infectious workup was negative, including serologies for HIV, hepatitis B and C viruses, Epstein-Barr virus, cytomegalovirus, syphilis, and *Borrelia burgdorferi*, as well as stool cultures for poliovirus and serum and stool botulinum toxin assays together with stool cultures for *Clostridium botulinum*, all of which were negative (Table 1).

In view of these findings, the diagnosis of GBS was established. The patient required intubation and mechanical ventilation because of an estimated vital capacity below 15 mL/kg and the absence of airway protective reflexes and received IVIG at a dose of 1 g/kg/day for two days. Due to the absence of clinical improvement, a second course of IVIG (1 g/kg/day for two days) was administered 10 days later, resulting in partial motor recovery. A third course was given 10 days thereafter (1 g/kg/day for two days), coinciding with significant neurological improvement, with axial muscle strength graded at 5/5 and limb strength at 3/5.

A tracheostomy was performed six days after admission because prolonged mechanical ventilation was anticipated. The patient was successfully weaned from mechanical ventilation on Day 50 of hospitalization and transferred to the general pediatric ward two days later. At the time of transfer, she was conscious and eupneic, with effective swallowing and cough and motor strength graded at 4/5 in all four limbs. At follow-up, three months after symptom onset, the patient had achieved complete neurological recovery, with a Hughes disability score of 2 compared with a score of 5 at admission.

## Discussion

Generally, the course of GBS is monophasic, and recurrence is rare, being described in only 3% of cases [5]. Recurrence is defined by the occurrence of two episodes meeting the criteria and separated by an interval greater than two months [6]. The clinical presentation of the second episode is usually similar to that of the first, which was not the case in our patient, in whom the direction of paralysis differed [5].

The classic presentation consists of acute, extensive, ascending, symmetrical paralysis evolving over less than four weeks, with cranial nerve involvement (facial paralysis and/or swallowing disorders), moderate sensory disturbances, and abolition of osteotendinous reflexes [7]. These elements may be absent at the onset, highlighting the importance of repeated clinical examinations [7]. This was the case in our patient, in whom cranial nerve involvement and abolition of tendon reflexes were absent at admission and appeared 24 hours later. Descending paralysis is observed in only 5% of cases and may cast doubt on the diagnosis. In such situations, paraclinical investigations are valuable in a disease in which the diagnosis is primarily clinical [7].

Electroneuromyography supports the diagnosis and helps identify the electrophysiological subtype, which has prognostic implications. However, findings may be normal early in the disease course; therefore, repeat testing may be necessary [7]. Lumbar puncture helps rule out infectious radiculitis (Lyme disease, varicella, and herpes zoster) and supports the diagnosis by demonstrating albuminocytologic dissociation. However, CSF findings may also be normal initially; albuminocytologic dissociation usually appears between the third and 15th day after symptom onset. This was the case in our patient, in whom albuminocytologic dissociation was detected only during the second lumbar puncture, performed nine days after symptom onset [7,8].

Spinal MRI helps rule out spinal cord lesions in cases of isolated lower-limb paralysis and/or marked sphincter dysfunction. Brainstem MRI is indicated in cases of cranial nerve involvement to exclude local lesions [7]. Measurement of anti-ganglioside antibodies is useful in atypical forms because positive results provide strong supportive evidence. In typical forms, however, their contribution is limited because sensitivity is variable and the turnaround time may range from a few days to several weeks [8].

Anti-ganglioside antibody results were obtained within nine days and provided additional support for the diagnosis. We compared our findings with the Brighton criteria (Table 2), a system developed in 2011 by international experts to standardize epidemiological and pharmacovigilance studies and considered useful in atypical presentations [8]. The patient fulfilled the criteria for the highest level of diagnostic certainty.

**Table 2:** Application of the Brighton Collaboration diagnostic criteria to the present case GBS, Guillain-Barré syndrome

Brighton criterion	Requirement	Patient findings	Criterion met
Bilateral and flaccid weakness of the limbs	Required	Bilateral upper limb weakness progressing to flaccid tetraplegia	Yes
Decreased or absent deep tendon reflexes in weak limbs	Required	Areflexia observed on neurological examination	Yes
Monophasic illness pattern and interval between onset and nadir of weakness between 12 hours and 28 days	Required	Progressive neurological deterioration over four days, reaching the nadir within the required timeframe	Yes
Absence of an alternative diagnosis explaining the weakness	Required	Extensive investigations, including infectious studies, neuroimaging, poliovirus testing, and botulinum toxin testing, failed to identify an alternative diagnosis	Yes
CSF findings consistent with GBS	CSF protein elevation with a cell count <50 cells/mm³	Initial CSF findings were normal; repeat lumbar puncture on Day 5 showed a protein level of 1.2 g/L with 0 cells/mm³ (albuminocytologic dissociation)	Yes
Nerve conduction studies consistent with GBS	Electrophysiological findings compatible with GBS	Electrophysiological studies demonstrated acute polyneuropathy consistent with GBS	Yes

A prodromal phase corresponding to a viral or bacterial infection often precedes GBS (approximately two-thirds of cases), and *Campylobacter *is responsible in 25-50% of cases. This phase is suggestive but not necessary for the diagnosis [7,9]. Our patient presented with an influenza-like syndrome with diarrhea 15 days earlier, probably of viral origin, given the benign nature of the presentation.

The main differential diagnoses of descending paralysis were systematically investigated and considered unlikely. Miller Fisher syndrome was excluded because of the absence of the characteristic clinical triad, particularly ophthalmoplegia and ataxia. Myasthenia gravis was also considered; however, there was no ptosis on clinical examination, and electrophysiological findings did not support this diagnosis. Botulism was ruled out owing to the absence of diplopia, ophthalmoplegia, and dysphonia, together with negative specific biological investigations. However, an overlap between the pharyngeal-cervical-brachial variant and classic GBS cannot be completely excluded, as these entities belong to the same clinical and immunological spectrum.

Treatment should be based on either IVIG or plasma exchange [10]. We opted for treatment with immunoglobulins because of their availability in the department and their favorable tolerability profile.

Regarding repeated courses of immunoglobulins after nonresponse to the initial course, this approach was adopted according to the European Federation of Neurological Societies guidelines, which allow administration of an additional course in patients who do not respond to the first. However, only one randomized controlled trial has evaluated the use of additional IVIG, with the remaining evidence consisting of case reports and observational studies. Currently, there is no evidence supporting repeat IVIG in patients with GBS who fail to improve after the initial course with respect to disability, mortality, the requirement for mechanical ventilation, or ICU admission [9,11]. Therefore, it cannot be determined whether the improvement observed after repeated courses was attributable to the additional treatment or to the spontaneous resolution of the disease.

## Conclusions

Although this report describes a single clinical case and therefore has limited epidemiological value, it provides an opportunity to emphasize the importance of considering and actively investigating GBS in cases of acute flaccid paralysis, even when the presentation is atypical, with a descending pattern of weakness and recurrent disease. Early recognition of such unusual presentations is essential to ensure timely diagnosis and appropriate management. Regarding the positivity of anti-GT1a and anti-GQ1b antibodies, any potential association with the descending pattern of paralysis observed in this case, reminiscent of that described in Miller Fisher syndrome and other anti-GQ1b spectrum disorders, remains speculative and warrants confirmation through larger-scale studies.
